# To Address Women's Health Inequity, It Must First Be Measured

**DOI:** 10.1089/heq.2022.0107

**Published:** 2022-12-02

**Authors:** Kathryn Godburn Schubert, Chloe E. Bird, Katy Kozhimmanil, Susan F. Wood

**Affiliations:** ^1^ Society for Women's Health Research, Washington, District of Columbia, USA.; ^2^ Tufts Medical Center, Boston, Massachusetts, USA.; ^3^ Tufts University School of Medicine, Boston, Massachusetts, USA.; ^4^ RAND Corporation, Santa Monica, California, USA.; ^5^ University of Minnesota School of Public Health, Twin Cities, Minnesota, USA.; ^6^ George Washington University Milken Institute School of Public Health, Washington, District of Columbia, USA.

**Keywords:** women's health, public health, data, health equity, public health data systems

## Abstract

Research and data collection related to what is historically known as “women's health” is consistently underfunded and marginalizes the health risks and experiences of women of color and transgender people. In the wake of the pandemic, the United States has an opportunity to redesign and reimagine a modern public health data infrastructure that centers equity and elevates the health and well-being of under-represented communities, including the full spectrum of gender identities. This piece offers a blueprint for transformational change in how the United States collects, interprets, and shares critical data to deliver greater health justice for all.

## Introduction

It is time to acknowledge that the United States needs a dramatic reset when it comes to “women's health” and how we measure it. For decades, our public health system has not only marginalized women's health but also viewed it through a flawed monolithic exclusionary lens. Research and data collection related to women's health have been consistently underfunded and excluded the health risks and lived experiences of important populations,^1^ such as women of color and transgender people. In this piece, we intend to instigate a conversation on the urgent need for transformational reform in the United States public health data infrastructure that centers equity by including sex assigned at birth, gender identity, race, ethnicity, and other statuses, to achieve more inclusive health policies.

The scarcity of real-time disaggregated data—across variables such as sex and gender identity, race, ethnicity, geographic location, age, and disability—contributed^2^ to the devastating toll of COVID-19 and systemic disparities in health outcomes^3^ among minority and marginalized populations. The lesson is clear: we cannot fix what we do not accurately measure. This country's data infrastructure must be fundamentally redesigned to center equity and elevate the health and well-being of marginalized populations.

To address our nation's enduring and chronic data failures, the Robert Wood Johnson Foundation^4^ created a National Commission to Transform Public Health Data Systems,^5^ with the goal of creating recommendations^6^ on how to build a modern equity-centered public health data infrastructure that helps policymakers identify problems, target interventions, and allocate resources to those most in need. Informing the Commission's deliberations were five expert panels, which examined population-specific data gaps that contribute to health inequities among American Indians/Alaska Natives, Black people/African Americans, lesbian, gay, bisexual, transgender, queer or questioning, intersex, asexual, and more (LGBTQ+) communities, people living with disabilities, and women.

The women expert panel^7^ was tasked with identifying the change needed in data systems to achieve greater sex and gender equity in health outcomes among women. Recognizing the intersectionality among population-specific groups, panelists were selected across race, ethnicity, sex, gender identity, lived experiences, geographic location and backgrounds in women's health research, policy, maternal, and public health. Collectively, participants represented perspectives from government and nongovernment sectors, and public and nonpublic health systems. Central to this expert panel's deliberations were in-depth discussions and analysis of the glaring gaps in women's health equity, across a span of intersecting identities, stemming from the historic lack of inclusion of women in health research and the dearth of accurate, disaggregated sex, and gender data.

We reference studies examining differences between women and men. Most include only cisgender people and/or were not clear as to whether transgender people were included. When we refer to “transgender people,” we mean transgender women and men. Furthermore, we acknowledge the consequences gender identity goes beyond how a person identifies, and includes multiple factors such as access to resources, roles and practices, norms, beliefs, decision-making power and autonomy, laws, policy, and institutions.^8^

## The Impact of Data Failures on Women's Health Equity

The pandemic provided a stark reminder of how the intersectionality of race, gender identity, and social determinants of health factor into a person's risk for poor health and disease-related outcomes. Although data found that more men than women^9^ in the United States died from the COVID-19 virus, critical variables such as race and line of work obscure the risk of death for women from certain communities.^*^ Studies in Georgia and Michigan showed the COVID death rate for Black women three times as high^10^ as that for White and Asian men, with disparities further exacerbated by the lack of uniform reporting of race/ethnicity in COVID-19 studies.^11^ Without examining the intersections of race and gender, researchers stated, “the blanket claim that women with COVID-19 fare better than men, makes invisible the high death rate among Black women.”^*^

Women hold 76%^12^ of the nation's health care jobs and account for the vast majority of frontline service workers,^13^ making them especially vulnerable to exposure. In addition, they are four times more likely^14^ to suffer from long COVID.^*^ Alongside longer lifespans^15^ and different comorbidities, the ramifications from COVID infections could potentially be more severe and costly for women over time. Without tracking and measuring these data points, we cannot fully understand who is at the greatest risk and the potential lifelong debilitating impacts.

Cisgender women—especially women of color—have been historically and contemporarily to be excluded from biomedical research. Only as recently as 2016 did the National Institutes of Health (NIH) institute a policy that sex as a biological variable be factored into its funded research. This policy aimed to ensure that differences according to sex assigned at birth are considered, but one unintended effect is erasure of transgender people in research. Research institutes and organizations continue to consistently underfund research^16^ on women, even when it comes to diseases that have the greatest impact on women's health. A recent analysis of NIH research funding patterns reveals there are roughly three times as many diseases^17^ as female-favored diseased in the NIH portfolio.

Furthermore, male-favored diseases are significantly more likely to be overfunded and female-favored diseases more likely to be underfunded.^*^ Nationwide, only one-third^18^ of cardiovascular disease clinical trial subjects are assigned female at birth, despite the fact that it is the number one killer of women in the United States.^*^ These glaring inequities are all the more irrational considering the immense societal return on investment that could be achieved through greater women's health research. Another study^19^ examining NIH's 2019 funding decisions revealed that a mere 4.5% of funding on coronary artery disease (CAD) addressed questions related to how the disease impacts women, but that doubling NIH funding for research on CAD in women would produce a return on investment of 9500% over 30 years.^*^

Gender, race, and age biases persist in clinical trials for life-saving vaccines and drugs. Although the FDA's 2020 Drug Trials Snapshots^20^ report shows that women made up >50% of drug trial participants, overall—among both women and men—only 8% of all trial participants were Black or African American, 11% were Hispanic, and 30% were over the age of 65. Pregnant people were excluded from COVID vaccine trials, leading to vaccine hesitancy^21^ among this population—even though global studies^22^ showed that they face significantly higher mortality rates from COVID-19. Unfortunately, it is difficult to determine the true extent of health inequities and impact of the pandemic due to the lack of data on minorities and marginalized populations, including cisgender women of color and transgender people.

## Standardizing Data Collection Around Sex and Gender Identity

Developing intentional consistent definitions of sex and gender data and establishing routine data collection are essential to identifying health disparities and achieving health equity. Data collection efforts must recognize evolving terminology related to the spectrum of gender identity, and the range of ways in which people—both cisgender and trans—experience health and the health care system, to capture a holistic understanding of health and its impact over time.

A 2022 report^23^ from the National Academies of Sciences, Engineering, and Medicine calls on the NIH to standardize language and practices used for data collection on sex and gender, “including collecting gender data by default, and not conflating gender with sex as a biological variable.” The report argues that, in most contexts, collecting data on gender is more relevant than collecting data on sex as a biological variable, and offers the following five guiding principles^24^ for data collection (Table 1).

**Table 1.** National Academies of Science, Engineering, and Medicine's Guiding Principles for data collection on sex, gender, and sexual orientation

Guiding principles for data collection on sex, gender, and sexual orientation	
	*Source: National Academies of Sciences, Engineering, and Medicine*
Inclusiveness	People deserve to be counted. Everyone should be able to see themselves and their identities represented in surveys and other data collection instruments.
Precision	Use precise terminology that reflects the complex and multidimensional nature of sex, gender, and sexual orientation.
Autonomy	Respect identity and autonomy. Data collection must allow respondents to self-identify whenever possible.
Parsimony	Collect only necessary data, gathered in pursuit of a specific and well-defined goal.
Privacy	Use data in a manner that benefits respondents and respects their privacy and confidentiality. Research findings should be shared with respondents to ensure the benefit, and data should be shared only under rigorous privacy and confidentiality standards.

As inclusive language practices evolve around sex and gender identity in the scientific and medical communities, we must proceed with intention on the use of language and definitions, to center equity in the approach to data collection, and to continually reassess definitions to ensure findings are correctly interpreted, shared, and compared across community-based and national data systems.

## Building Women's Health Equity Through Data Systems Transformation

Research and data on women's health are inextricably linked to many facets of identity and other social determinants, impacting health outcomes. Yet our outdated data systems are ill-equipped to capture this vital information. Health care systems that are designed to routinely collect and report a standardized and comprehensive set of essential data points must be structured to allow for intersectional analysis between variables and remain nimble as data are collected over time. The ability to pull data by sex or gender identity, as well as by race, ethnicity, disability, employment, and socioeconomic status, and other variables, will help identify the biggest health gaps and prioritize areas of focus.

After thoughtful deliberations, the women expert panel reached unanimous agreement on four overarching recommendations and calls-to-action to reinvent America's public health data systems, with an eye toward women's health equity (Table 2).

**Table 2.** Recommendations and Calls-to-Action

Recommendation 1: Standardization of essential data points	
Rationale	Calls-to-action
It is imperative that we build a new public health data infrastructure cemented in a foundation of standardized, comprehensive essential data points to identify women's health inequities across one's “life course” and help shape policies to address disparities. The standardization of data collection best practices will facilitate intersectional analysis between variables, such as sex and gender identity, and other social determinants impacting health outcomes. Also critical is the standardization of definitions being used in the collection of sex and gender identity data to ensure a complete and accurate understanding of health inequities across the spectrum of sex and gender.	Agree on a set of guidelines, goals, objectives, measurable outcomes, and benchmarks for success. Create and adopt universal data elements and standards—including consistent standardized definitions of sex and gender identity—within an overarching data governance structure applied across data systems. Ensure adequate sustainable funding for standardized data collection, management, integration, collaboration, reporting, and evaluation.

Recommendation 2: interoperability and interconnectivity of data and systems	
Rationale	Calls-to-action
America's siloed disconnected health care data systems have made it extremely difficult to measure women's health data in a holistic impactful way. Currently, the lack of interoperability among systems makes it largely impossible to integrate social determinants of health into data gathering processes. Interoperability between data systems stymies the critical sharing and analysis of disaggregated health data across platforms and communities. This, in turn, prevents women from receiving preventive quality care and treatment, especially among those most at risk for developing serious illness and disease.	Establish interoperability and ability to overlay or connect data sets to support longitudinal overtime analysis. Prioritize high-value data sets and systems for conformity and interoperability. Identify specific gender-based disparities and how gender discrimination interacts with other oppressions, and targeted data connectivity initiatives to address them. Advocate for policy change to address barriers to data sharing and interoperability.

Recommendation 3: longitudinal tracking for lifespan measurement	
Rationale	Calls-to-action
Health data captured and linked over time and across an individual's lifespan will improve long-term health outcomes for women. In rebuilding America's data infrastructure, it is critical that our data systems have the capability of interacting throughout one's health journey. The health transitions traditionally experienced by cisgender women are also important for transgender people across their lives—from pediatric to adult care, preconception into pregnancy and postpartum for those of childbearing capabilities, and finally, life stage transitions such as menopause through to Medicare—allow us to identify data linkage failures, the lack of collection of multiple data points, and other gaps and areas of vital need in women's health.	Develop a long-term roadmap and timelines for milestones for implementation of health data transformation with an eye toward lifespan measurement and strengthening women's health equity. Implement a strategy for tracking and addressing data gaps that affect women's health equity over time. Make integrated “lifespan” data accessible for a wide range of stakeholders to examine and assess progress in women's health care and equity over time.

Recommendation 4: multisector buy-in for data systems transformation	
Rationale	Calls-to-action
It is essential that we build the diverse multisector stakeholder buy-in required to champion the transformation of America's public health data systems, with a focus on women's health equity. Individuals and organizations from health care delivery, financing, patient and consumer advocacy, research, and regulatory sectors should be involved in developing and implementing a comprehensive approach to improving data integrity and interconnectedness across the health care landscape. Multisector collaboration will ensure public and private data collection systems adhere to standardized best practices to collect critical health variables that include both biological sex and gender identity, and other social determinants such as race/ethnicity, to ensure intersectional impacts can be accurately identified and addressed.	Assess funding needs and opportunities and develop a cost-benefit analysis to support funding requests across sectors. Integrate a wide-ranging set of stakeholders, including advocates for high-risk populations impacted by systemic health inequities. Raise awareness across sectors on health disparities experienced by women and the important role of data in correcting these inequities.

## Conclusion

Now is the time to widen our lens on “women's health” to include research and data across the full spectrum of gender identities, sex, race, ethnicity, and other variables to address systemic health disparities. The United States has an unprecedented opportunity—and obligation—to rebuild a modern public health data infrastructure, cemented in a foundation of equity and inclusion. The recommendations outlined earlier by the women expert panel on population-specific data gaps, convened by the Robert Wood Johnson Foundation, offer a blueprint for transformational change in how the United States collects, interprets, and shares critical data on sex and gender that is inclusive of all numerical gender minorities. This disruptive model for data systems reform has enormous potential to transform care, enhance equity, and create empowerment through better health outcomes for generations to come.

## Abbreviations Used

CAD: coronary artery disease
NIH: National Institutes of Health

## Notes

^*^
This research article uses the term “men” and “women” although they do not indicate whether this was inclusive of trans men, trans women or only cis men and cis women.
